# Advances in Kinetic Isotope Effect Measurement Techniques for Enzyme Mechanism Study

**DOI:** 10.3390/molecules18089278

**Published:** 2013-08-02

**Authors:** Hong Gu, Shuming Zhang

**Affiliations:** Department of Biochemistry, Case Western Reserve University School of Medicine, 10900 Euclid Ave., Cleveland, OH 44106, USA; E-Mail: sxz157@case.edu

**Keywords:** kinetic isotope effects, measurement, mass spectrometry, nuclear magnetic resonance, liquid scintillation counting

## Abstract

Kinetic isotope effects (KIEs) are a very powerful tool for investigating enzyme mechanisms. Precision of measurement is the most important factor for KIE determinations, especially for small heavy atom KIEs. Internal competition is commonly used to measure small KIEs on V/K. Several methods, including such as liquid scintillation counting, mass spectrometry, nuclear magnetic resonance spectroscopy and polarimetry have been used to determine KIEs. In this paper, which does not aspire to be an exhaustive review, we briefly review different experimental approaches for the measurement of KIEs on enzymatic reaction with an emphasis on newer techniques employing mass spectrometry and nuclear magnetic resonance spectrometry as well as some corresponding examples.

## 1. Introduction

**Scheme 1:**
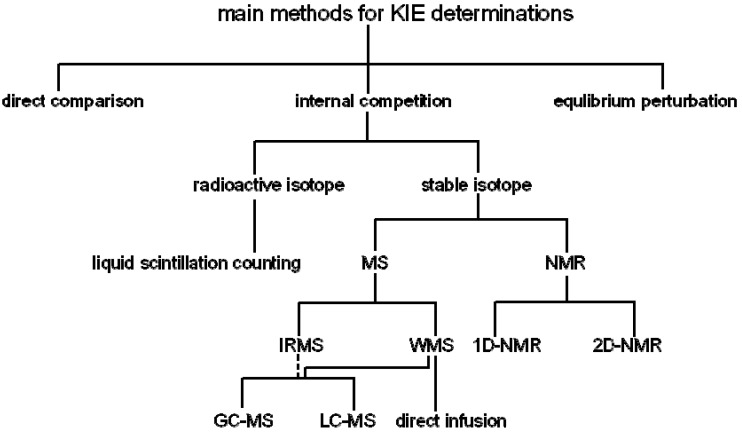
The internal competition method for KIE determination are divided between radioactive isotope, such as C-14 (^14^C) or H-3 (^3^H), and stable isotopes, such as O-18 (^18^O), N-15 (^15^N) and C-13 (^13^C). IRMS, isotope ratio mass spectrometry; WMS, whole molecular mass spectrometry; NMR, nuclear magnetic resonance; 1D-NMR, one dimension NMR; 2D-NMR, two dimension NMR; GC-MS, gas chromatography coupled to mass spectrometry; LC-MS, liquid chromatography coupled to mass spectrometry.

Kinetic isotope effects (KIEs) are an essential tool that has been developed to understand catalytic mechanisms and/or elucidate the transition states in chemical and enzymatic reactions. [1,2,3,4,5,6,7,8,9,10,11] Primary KIEs occur when a bond is formed or broken to the isotopic atom during the reaction while secondary KIEs are observed when the number of bonds to the isotopic atom remains unchanged. In theory, the KIE is caused by the difference of vibrational frequencies for the light (nature) and heavy (isotope) atoms (molecules), and their associated zero point energies, at ground and transition states. Over the last 50 years, a number of different approaches to the direct measurement of KIEs in enzymatic reactions have been employed. The direct comparison, equilibrium perturbation and the internal competition method are the three main methods for the measurement of KIEs as introduced in detail by Cleland [9,12]. The precision of the internal competition method has made it the most commonly used for investigating small KIEs, which is limited for technical reasons to the determination of KIEs on V/K. If KIEs are to be determined on V, a direct comparison method is required [13,14]. A major advantage of the competitive method is that it avoids systematic errors by measuring changes in the isotope ratio of the two isotopologues. Both radioactive isotopes and stable isotopes have been used for the competitive method, and mass spectrometric (MS) and nuclear magnetic resonance (NMR) spectrometric approaches are applied to determine the isotope ratio of light and heavy atoms (Scheme 1). Stable isotopes have advantages over radioactive isotopes. The synthesis of substrates with stable isotopes is easier than the incorporation of radioactive isotope labels and the former can avoid the handling of radioactive samples with their potential for harm and regulatory burden. In general, precision is the most important factor for the KIE measurement especially for small heavy atom KIEs.

Although Paneth [15] summarized some analytical methods for heavy atom kinetic isotope effects measurements in 1987; the major focus of this review is on the use of advanced techniques, in particular recent improvements in high resolution of mass spectrometry and high field nuclear magnetic resonance spectrometry that have enabled precise isotope ratio determinations, as well as some new aspects of the radioactive competitive method for the measurement of KIEs on enzymatic reactions.

## 2. Measurement of KIEs by Mass Spectrometry

Coupling stable isotopic labeling of substrates with the competitive method for the measurement of KIEs by mass spectrometry has employed two different kinds of mass spectrometry: isotope-ratio mass spectrometry (IRMS) and whole (routine) molecule mass spectrometry (WMS). IRMS allows superior precise measurements of mixtures of stable isotopes [16,17,18,19]. Several separation techniques have been coupled to an IRMS, which are liquid chromatographic (LC), gas chromatographic (GC), and elemental analyzers (EA), but actually only EA and GC are available for commercial IRMS analyses. IRMS has inherent advantages, such as the high precision on the order of 0.01% that can be obtained (in general in the range of 4–6 significant figures). However, IRMS is limited to be only useful for small gaseous molecules such as N_2_, H_2_, CO, NO, N_2_O, SO_2_ or CO_2_, or to analytes whose isotopically labeled atom can be uniquely and quantitatively converted to one of these analytes; thus this method was restricted to a limited class of enzymes, such as decarboxylases and deaminases. To overcome this limitation, Cleland and coauthors introduced for the first time a remote label method reported by O’Leary and coauthors [20] coupled with IRMS which can be used for determining KIEs for a variety of enzymatic reactions with high precision. Both stable isotopes such as C-13 (^13^C), N-15 (^15^N), and O-18 (^18^O) and radioactive isotopes such as C-14 (^14^C) can adapted to the remote label method. Hermes and Cleland [21] have used the remote label method with IRMS for a series of thorough studies of ^18^O KIE and other isotope effect studies on the glucose-6-phosphate (G6P) dehydrogenase reaction, and have also applied this method to great effect to investigate the transition states of the reactions catalyzed by enzymes such as adenosine deaminase and aspartate transcarbamylase [22,23,24,25]. It is critical that the mixture of unlabeled and isotope labeled molecules be purified when the remote label method is used. An enriched contaminant that does not react and a depleted contaminant that is already in the product form will generate artifactual isotope effects, in general they will give higher results. The use of WMS technique can overcome this limitation for the remote label method. 

**Scheme 2:**
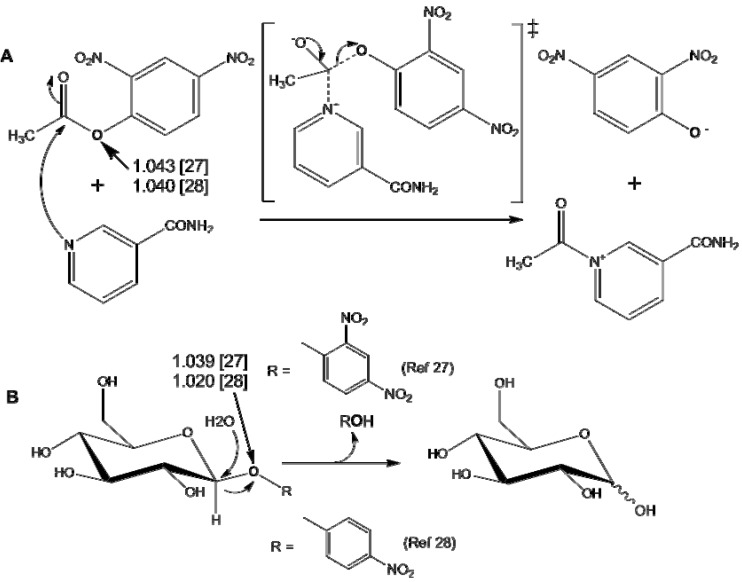
The mechanisms along with the observed KIEs for the aminolysis of 2,4-dinitrophenyl acetate (DNPA) (**A**) and the hydrolysis of galactosides (**B**) reported in references [27,28].

**Scheme 3:**
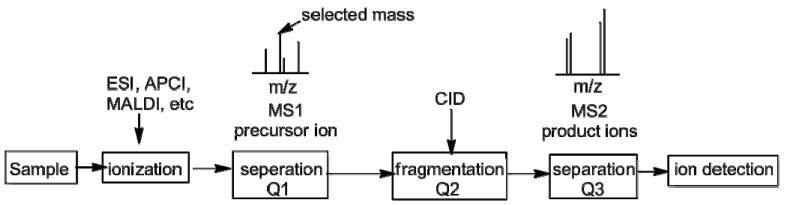
Tandem mass spectrometer. ESI, electrospray ionization; APCI, atmospheric pressure chemical ionization; MALDI, matrix-assisted laser desorption ionization; CID, collision-induced dissociation. Product ions cam apply select ion monitoring (SIM) and multiple reactions monitoring (MRM).

The WMS method was first introduced to determine KIEs in 1978 by Cooks and coauthors [26] who reported that the chlorine isotope effects (*k*^35^Cl/*k*^37^Cl) for reactions dealing with Cl·and HCl elimination were unity to 1.20 with an error of less than 5%. This determination employed a mass-analyzed ion kinetic (MIKE) spectrometer by multiple reaction monitoring. Then Rosenberg and Kirsch [27,28] also reported in 1979 that the heavy oxygen-18 kinetic isotope effect, ^18^O-KIE, for the leaving group in the aminolysis of 2,4-dinitrophenyl acetate (DNPA) by nicotinamide (see Scheme 2A) determined directly by analyzing the parent ion following electron impact ionization in a mass spectrometer in two independent experiments was 1.043 ± 0.007 [27] and 1.040 ± 0.002 [28], respectively with the standard errors of 0.2%. They also reported that the observed ^18^O-KIEs with direct mass spectrometric analysis for the leaving groups 2,4-dinitrophenol and *p*-nitrophenol, in the hydrolysis of 2,4-dinitrophenyl-β-D-galactoside and *p*-nitrophenyl-β-galactoside by β-galactosidase from *E. coli* were 1.039 ± 0.006 [27] and 1.0200 ± 0.007 [28], respectively (Scheme 2B). This precision is sufficient for the determination and interpretation of KIEs for ^13^C, ^15^N, and ^18^O.However, this method has infrequently applied due to the signal instability and background contamination. Fortunately, with recent improvements in the sensitivity of mass spectrometry and new ionization methods, stable isotopic-labeling coupled with electrospray ionization (ESI)-tandem mass spectrometry (MS/MS) (Scheme 3) can be used for the measurement of KIEs by the competitive method with enhanced precision to yield meaningful results [29,30,31]. By the early 1990s, the study reported by Anderson and coauthors [32,33] showed that WMS can be applied to measure the enzymatic catalyzed KIEs with a precision similar to the radioactive-labeled method. The method developed by Anderson and coauthors [32,33] was further confirmed by Schramm and coauthors [34] who reported that the ring oxygen, 4′-^18^O KIE of NAD^+^ hydrolysis catalyzed by diphtheria toxin measured by whole molecule mass spectrometry is 0.991 ± 0.003, while it is 0.986 ± 0.003 with the radioactive label method. The KIE measured by the two different methods agree within the reported experimental errors. Cassano, Anderson, and Harris [35,36] further reported the solvent nucleophile isotope effects (^18^*k*_nuc_) for hydroxide attack on T5-PNPP (thymidine-5′-*p*-nitrophenyl phosphate) and on pre-tRNA to yield pGp determined by ESI-MS via 321/323 ion pair (product of reaction, thymidine-5′-monophosphate, TMP) and 442/444 ion pair with single ion monitoring (SIM) which generates significantly greater sensitivity and signal/noise ratio than normal acquisitions. Anderson and coauthors [37] also discussed in detail the inaccuracies in SIM determination of isotope ratio.

**Figure 1:**
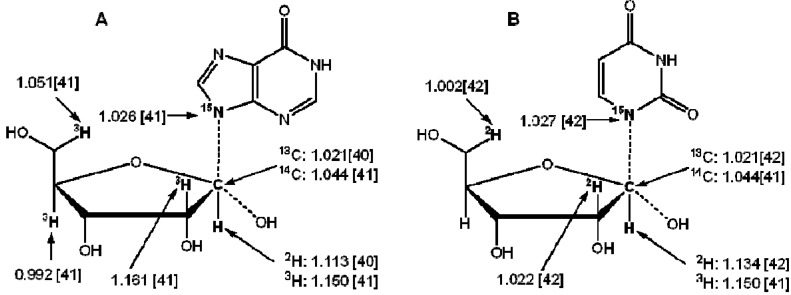
Observed KIEs of inosine (**A**) and uridine (**B**) for the enzymatic catalysis determined with GC-MS, LC-MS and radioactive-labeled methods [40,41,42].

When the WMS is applied to determine KIEs, two different methods of sample introduction have been used: direct infusion and GC- or LC-coupled MS (GC-MS or LC-MS). Direct infusion means a sample containing a mixture of unlabeled and labeled molecules is continuously introduced into the MS for ionization and analysis.. This procedure has the potential of enhanced precision obtained from extended times of data acquisition. Depending on background and salt concentrations, the substrate/product to be analyzed may need to be purified from the reaction mixture. Using a direct transfer from a GC or LC in the GC-MS or LC-MS implementation provides the required sample separation, enabling the direct use of the quenched the mixture without prior purification and avoiding use of the reaction mixture could excessively contaminate the ion source. The MS can analyze either the molecular ion or if an appropriate fragment ion is identified, tandem mass spectrometry can be used to improve signal/noise. The higher resolution available with Q-TOF instruments, as compared to triple quadrupoles has provided more reliable and accurate isotope ratio determinations. Kakinuma and coauthors [38] reported that the ^2^H KIE of 2-deoxy-*scyllo*-inosose (*k*_H_/*k*_D_) catalyzed by 2-deoxy-*scyllo*-inosose synthase derived from *Bacillus circulans* determined by GC-MS was 2.4 which is the same KIE range (2.6) for the same enzyme derived from *Streptomyces fradiae* [39]. Lee and coauthors [40] also reported the KIEs of [1′-^13^C]inosine and [1′-^2^H]inosine catalyzed by nucleoside hydrolase from *C. fasciculate* determined by GC-MS under selected ion monitoring (SIM) condition at three ion pairs (158/159, 187/188, and 217/218) were 1.021 ± 0.006 and 1.113 ± 0.008, respectively and the transition state is consistent with that reported by Schramm and coauthors [41] using radioactive substrates which means GC-MS is reliable to determine KIEs (Figure 1A). Kline and coauthors [42] determined KIEs of [1′-^13^C], [1-^15^N], [1′-^2^H], [2′-^2^H] and [5′-^2^H_2_]uridine by nucleoside hydrolase from *E. coli* with LC-MS under the SIM conditions analyzing the 173/174 and 113/114 ion pairs for the ribose and uracil moiety, respectively and the elucidated transition state is consistent with a bond-energy bond-order vibrational analysis (Figure 1B). The KIE of [1-^15^N]uridine determined by LC-MS is almost the same as the KIE of [1-^15^N]inosine measured with scintillation counting via radioactive labeled nucleosides reported by Schramm and coauthors [41]. The KIEs of [1′-^13^C]inosine and [1′-^13^C]uridine for *E. coli* nucleoside hydrolase are same (1.021) as measured by GC-MS [40] and LC-MS [42], respectively. These values indicate that the C1′-N1 bond is broken in the transition state. Pollack and coauthors [43] analyzed α-secondary deuterium KIEs of Δ^5^-anfrostene-3,17-dione at C-6 catalyzed by ketosteroid isomerase and acetate with direct injection ESI-MS via the ion pair of 287/288 giving the KIE of 1.073 and 1.031, respectively, which suggested the possibility of coupled motion/hydrogen tunneling in both enzymatic and non-enzymatic systems. The secondary ^2^H KIEs for the active site mutants of ketosteroid isomerase are similar to that of wild type [43]. While Yamashita and coauthors [44] determined the ^2^H KIE (*k*_H_/*k*_D_ = 0.97) of [19,19-^2^H_2_]19-hydroxy-6-oxo-androstenedione (19-ol-6-oxoAD) catalyzed by placental aromatase with LC-MS (ESI) (375/377 ion pair).

**Figure 2:**
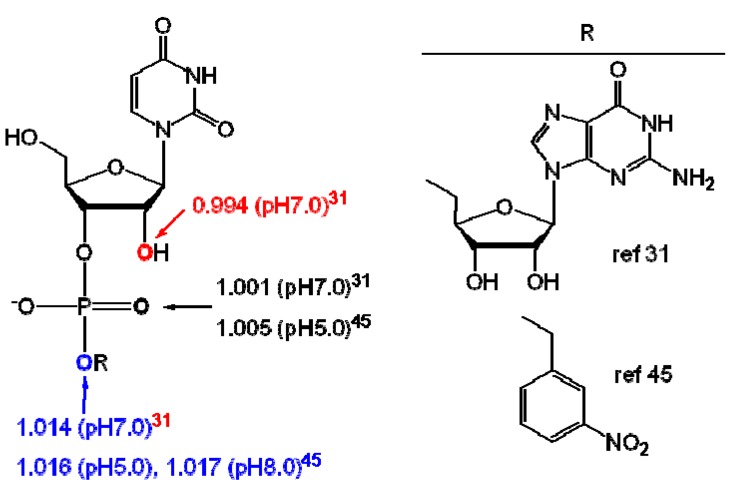
Observed KIEs for UpG and 3′-m-nitrobenzyl phosphate catalyzed by RNase A measure by WMS and IRMS, respectively [31,45].

Lately, using the direct infusion approach we measured the ^18^O KIEs to characterize the mechanism of phosphotransferase reactions promoted by both chemical [29,30] and enzyme (ribonuclease A) [31] catalysts using competitive stable isotope labels coupled with nano-ESI-MS/MS. In 1997, Sowa, Hengge and Cleland [45] reported ^18^O KIEs on the ribonuclease A catalyzed reaction using the substrate uridine 3′-*m*-nitrobenzyl phosphate with a competitive remote label method coupled to IRMS. The KIE for the leaving group (LG) oxygen, ^18^(V/K)_LG_, was 1.016 ± 0.001 (pH 5.0) and 1.017 ± 0.001 (pH 8.0), while the KIE on the nonbridging phosphoryl oxygens (NPO), ^18^(V/K)_NPO_, was 1.005 ± 0.001 at pH 5.0 [45]. Using the WMS method, we determined the ^18^O KIEs on the ribonuclease A catalyzed reaction using a more physiologically relevant substrate, uridylyl-(3′,5′)-guanosine (UpG), and found the KIEs on the leaving group [^18^(V/K)LG], non-bridging phosphoryl [^18^(V/K)NPO], and nucleophilic oxygen [^18^(V/K)NUC] determined at pH 7.0 were 1.014 ± 0.003, 1.001 ± 0.001, and 0.994 ± 0.002, respectively [31]. The KIEs measured by two different methods agree within experimental error (Figure 2). This shows that the KIE measured by WMS [31] are consistent with the KIEs determined by IRMS [45].

Our newly developed approach overcomes two experimental challenges: first synthesis of site-specific isotopically enriched RNAs with ^18^O, and second monitoring RNA isotopic composition with high precision. The analytical challenge is overcome by using nano-ESI-Q/TOF MS/MS. This method allows for the determination of the change in the ratio of two isotopologues, simultaneously, throughout the whole reaction course, *i.e.*, as a function of the fraction of reaction. To calculate the KIE, the change in the ratio of the two substrate isotopologues is obtained from the initial composition of 5% to 95% reaction completion, five time points were taken. The use of nano-ESI-Q/TOF MS/MS, overcomes the two limitations: the sensitivity is very high and by using tandem mass spectrometry, the signal-to-noise ratio is high enough thus background is negligible. The *m/z* resolution of TOF not only gives base line resolution, but can resolve contaminants with the same nominal *m/z*. All of these features contribute to the precision and accuracy of the measurements. 

More recently, Wilson and coauthors [46] used a new straightforward approach based on Time-Resolved ElectroSpray Ionization Mass Spectrometry (TRESI-MS) to measure KIEs direct from the enzymatic reactions. TRESI-MS is an electrospray-coupled rapid mixing technique that enables monitoring of solution phase processes on the ms time-scale [46]. The author first determined the well-studied KIE (2.19 ± 0.05) in the Yeast Alcohol Dehydrogenase (YADH)-catalyzed hydride transfer from ethanol to NAD^+^, and then measured a small primary ^12^C/^13^C isotope effect (1.09 ± 0.02) on the *p*-nitrophenyl acetate (*p*NPA). This approach may also potentially apply to investigate enzymatic mechanisms and transition states, but the peak overlap and accuracy should be overcome for KIE determinations.

## 3. Measurement of KIEs by Nuclear Magnetic Resonance Spectrometry

**Figure 3:**
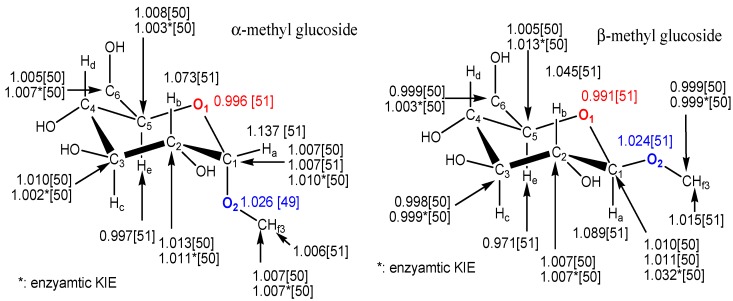
Observed ^2^H-, ^13^C-, and ^18^O-KIEs for methyl glucosides measured by NMR spectroscopy extracted from refs [50,51].

The use of the competitive method for the measurement of KIEs by nuclear magnetic resonance spectrometry (NMR) was reported as early as 1986 by Pascal *et al*. [47] who measured deuterium kinetic isotope effects (^2^H KIE) in organic and biological reactions by natural abundance NMR. In theory, all NMR-active nuclei can be determined at natural abundances that can be used to calculate KIEs; but it is difficult to quantify accurately their abundances using early NMR instruments due to their low resolution and precision, and consequently in practice it is hard to accurately measure the small KIEs because of the very low natural isotope abundances. Furthermore, NMR can’t be directly used to measure ^18^O KIEs since it lacks a nuclear spin. Using NMR at natural abundance to determine KIEs can avoid the synthesis of isotopically enriched substrates, more accurate and precise KIE measurements are possible by NMR if stable isotope labeled substrates are used. In 1995, Singleton and Thomas [48] reported multiple small KIE measurements at natural abundance with ^2^H- and ^13^C-NMR for isoprene. The average ^2^H KIE for the 1 and 4 positions of isoprene is 0.942 [48] that is very close to the value (0.935 to 0.948) previously reported by Gajewski *et al*. who measured them with GC/MS employing chemical ionization (CI) mode or capillary GC [49]. Lee, Bain, and Berti [50] also reported the KIEs of α- and β-methyl glycoside on the acid- and enzymatic-catalyzed hydrolysis by NMR at natural abundance. The results are summarized in Figure 3 along with the data generated from others. The acid-catalyzed ^13^C KIEs are excellent agreement with Bennet and Sinnott [51] who also determined ^18^O KIEs. Recently, Pratt and Crich as well as their coauthors presented the primary ^13^C KIEs for the products of a class of chemical glycosylation (α- and β-mannopyranosides and α- and β-glycopyranosides) using a natural abundance NMR method [52]. Their ^13^C KIEs are 1.005, 1.023, 1.023, and 1.019, respectively, with reasonable standard deviations. The approach for determining KIEs by nuclear magnetic resonance spectrometry at natural abundance was limited by the low sensitivity, and consequently the requirement for larger amounts of substrate and/or products to be analyzed. However, the instrument sensitivity of nuclear magnetic resonance spectroscopy has been dramatically improved by increases in field strength and the introduction of cryoprobes. Bennet and coauthors [53,54] developed a useful protocol to improve both the sensitivity and precision of the isotope effect measurement by direct nuclear magnetic resonance spectroscopy. They synthesized ^13^C- and ^18^O-labeled materials. These stable isotope labeled materials along with the wild type materials allow the acquisition of isotopologue ratio data continuously and avoid the need for the larger quantities of material that would be required for natural abundance measurements. Previously, only Cleland’s isotopic equilibrium perturbation method and our newly developed method allowed for continuous data acquisition. This new approach developed by Bennet and coauthors [53] also has advantages as it obviates the need for an internal reference atom, which is required for the current NMR and radioisotope methods. Chan and Bennet [54] also use the splitting of the ^19^F-NMR signal for KIE determinations. A stepwise solvent-promoted S_N_1 reaction of α-D-glucopyranosyl fluoride: mechanistic implications for retaining glycosyltransferases. In 2011, Pabis and Paneth [55] modified Bennet’s method to increase sensitivity by using ^1^H-NMR to measure ^13^C KIEs. They used a combination of two isotopologues (^12^C and ^13^C), from the ^1^H-NMR spectrum, to obtain the ^13^C/^12^C ratio. The advantage of this method is an increase in the sensitivity of the measurement by using ^1^H-NMR spectrum, instead of ^13^C-NMR [55].

More recently, Murkin and coauthors [56] reported a new method, ^1^H-detected 2D [^13^C,^1^H]-heteronuclear single quantum coherence (HSQC) NMR spectrometry, to determine KIEs. This method measure KIEs with high accuracy and precision. The 1-^13^C KIE of G6P for *Leuconostoc mesenteroides* G6P dehydrogenase determined by 2D-[^13^C,^1^H]-HSQC is 1.0172, in good agreement with the value of 1.0165 reported by Hermes and coauthors with IRMS [22]. The experimental errors of 2D HSQC for the ^13^C KIEs is very small (0.02%–0.12%) [56] that is very close to the error by IRMS. Furthermore, 2D HSQC is not required for additional sample manipulation and is useful for measuring small KIEs such as ^15^N and ^18^O.

## 4. KIE Calculations from Isotope Ratios and Their Uncertainties

The KIEs from the reaction substrate or product can be calculated with the following standard equations if the labeled (heavy) substrate is a competitive inhibitor of the unlabeled (light) one and *vice versa* [6]:

Based on the residual substrate:
*KIE*_calc_ = 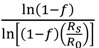
(1)
where *f* is the fraction of reaction, *R*_0_ is the isotope ratio of staring (initial) substrate, and *R*_s_ is the isotope ratio of unreacted (residual) substrate at fraction of reaction. Then the uncertainties at fraction of reaction (*f*) and the isotope ratio of light and heavy atoms (corresponding molecules) (*R*_s_/*R*_0_) can be expressed with:

Δ*KIE_f_* = 
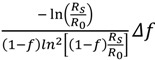
 (*f* is variable)
(2)
and:

Δ*KIE_R_* = 
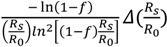
 (*R*_s_/*R*_0_ is variable)
(3)

Based on the observation of reaction product:
*KIE*_calc_ = 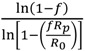
(4)
where *f* is the fraction of reaction, *R*_0_ and *R*_p_ are the isotope ratio of the starting substrate and the product at fraction of reaction, respectively. Their uncertainties at *f* and the isotope ratio of light and heavy atoms (corresponding molecules) (*R*_p_/*R*_0_) can be expressed with:

Δ*KIE_f_* = 
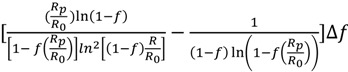
 (*f* is variable)
(5)
and:

Δ*KIE_R_* = 
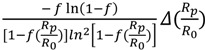
 (*R*_p_/*R*_0_ is variable)
(6)

For both on the substrate and product:
*KIE*_calc_ = 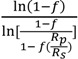
(7)

**Figure 4:**
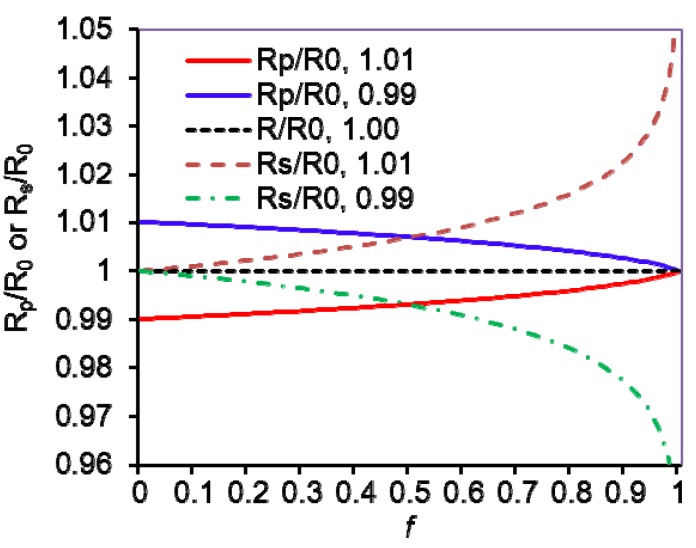
Simulated isotope ratios in product (*R*_p_) and remaining substrate (*R*_s_) to the initio isotope ratio (*R*_0_) in the substrate (*R*_0_) versus a function of fraction reaction *f* and at KIE (V/K) = 1.01 and 0.99.

Figure 4 shows the simulated isotope ratios of *R*_p_/*R*_0_ and *R*_s_/*R*_0_ at 1.01 and 0.99 of KIE by Equations (1) and (4), which indicates that the smaller fraction of reaction should be the one used when it is measured from product while the larger one should be used when it is measured from the remaining substrate. It is more sensitive for the error of *f* to ΔKIE than the error of isotope ratios in *R*_s_/*R*_0_ when *f* is over 0.5, in contrast ΔKIE is more sensitive to the error of *R*_p_/*R*_0_ than the error of *f*.

IRMS and radioactive isotope ratio determinations have employed trace labels. Consequently when measuring the reaction fraction, only the reaction of the light isotope was considered. In the WMS and NMR methods discussed, enriched substrates have been employed and the chemical determination of the fraction of reaction consequently has to include reaction of both isotopologues. Cleland [9,57] has solved the differential equations and provided the correct general formulas for both analyses of the isotope ratio in the products or in the residual substrate:
*Product measurement KIE* =
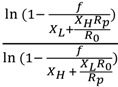
(8)
*Residual substrate KIE* = 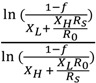
(9)
where KIE is the V/K isotope effect of (V/K)_L_/(V/K)_H_; the L and H stand for the two different isotopes, in general unlabeled (light, L) and labeled (heavy, H) atoms (molecules); *X_L_* and *X_H_* are the initial mole fractions of the light and heavy isotope substrates, respectively; *R*_s_ and *R*_p_ are the determined isotope ratio of L/H in the residual substrates and in the products at fractional reaction *f*, respectively; and *R*_0_ is the initial isotope ratio *X_L_*/*X_H_*. In detail, *f* = 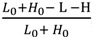, *R*_s_ = 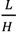, *R*_p_ = 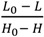, and *R*_0_ = 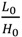 =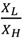; where *H_0_* and *L_0_*, *H* and *L* represent the concentration of the heavy and light substrates at initio and the fractial reaction, *f*, respectively.

## 5. Measurement of KIEs by Liquid Scintillation Counting

The measurement of KIEs by liquid scintillation counting routinely achieves a high precision of ±0.3% [58]. Schramm for the first time used ^14^C or tritium as remote labels in his work on *N*-ribosyltransferases and has subsequently applied this radioactive competitive method in a series of elegant studies on different enzymes [2,3,59,60]: AMP nucleosidase, thymidine phosphorylase, human and bovine purine nucleoside phosphorylase, human 5′-methylthioadenosine, bacterial methylthio-adenosine/adenosylhomocysteine nucleosidase hydrolases, ricin A chain and saporin. Scintillation counting has been widely applied to analyze KIEs, but it needs radioactive isotopes which will restrict its applications due to the safety issues. This method now is mainly used to analyze hydrogen tunneling by comparing ^1^H/^3^H and ^2^H/^3^H isotope effects and heavy atom KIEs via competitive methods.

When using the radioactive isotope method, first, the labeled substrates need to be synthesized; and careful purification of the labeled substrates is needed to remove any labeled contaminants. Because the liquid scintillation counting detects all radioactive labeled compounds, whether they are labeled substrates, labeled products, or any labeled intermediates, any labeled contaminants can introduce large errors in the calculated KIE. For example, when measuring KIEs by the ratio of ^14^C/^3^H in the product, the presence of 0.2% contaminants can generate over 1% errors in the calculated KIE [58].

Berti, Blanke, and Schramm [34] reported the transition state structure for the hydrolysis of NAD^+^ catalyzed by diphtheria toxin. In this paper, they discussed that WMS allows the determination of the isotopologue ratio directly; whereas using liquid scintillation counting to determine the KIEs by dual-radiolabel competitive method is an indirect measurement. However, with proper care, this method generates satisfactory accuracy for KIE investigations. Human and bovine purine nucleoside phosphorylases are the best examples for the determination of KIEs by the dual-radiolabel competitive method. Schramm and coauthors [59,60] reported that human and bovine purine nucleoside phosphorylase have different KIE values and hence stabilize different transition states.

Kilinman and coauthors [61] have used the radioactive competitive method to measure the KIEs of thermophilic alcohol dehydrogenase and soybean lipoxygenase-1, for the study of enzymatic H transfer reactions. Kohen and coauthors [62] have also used the radioisotope (^3^H) and IRMS competitive methods to measure the KIEs of ^1^H, ^2^H, and ^3^H on chromosomal, *fol*A-encoded dihydrofolate reductase (cDHFR) and the R plasmid-encoded R67 DHFR from *E. coli*.

There are numerous advantages for the measurement of KIEs by these radioactive isotope methods, including high precision and the requirement that only tracer levels of radioactive materials needed. Using this method, with proper attention and controls, errors from radioactive contaminants can be avoided. 

## 6. Conclusions

The substantial improvement in sensitivity and resolution available from the new generation of mass spectrometers (MS) and nuclear magnetic resonance (NMR) spectrometers has enhanced the capacity of experimentally quantitative determination of KIEs. These methods obviate the need to synthesize the radioactive labeled materials but just to synthesize the stable isotope labeled materials for KIE measurements by both MS and NMR. The improvement of these techniques will provide more accurate and precise for determining small KIEs. Experimental improvements open up new possibilities to elevate our knowledge of the transition state structure of enzyme catalyzed reactions.
